# Analysis of Diffracted Mode Outcoupling in the Context of Amplified Spontaneous Emission of Organic Thin Films

**DOI:** 10.3390/polym16131950

**Published:** 2024-07-08

**Authors:** Thilo Pudleiner, Jan Hoinkis, Christian Karnutsch

**Affiliations:** 1 Research Group Integrated Optofluidics and Nanophotonics (IONAS), Institute for Sensor and Information Systems, University of Applied Sciences Karlsruhe, 76133 Karlsruhe, Germany; thilo.pudleiner@h-ka.de; 2 Institute for Sensor and Information Systems, University of Applied Sciences Karlsruhe, 76133 Karlsruhe, Germany; jan.hoinkis@h-ka.de

**Keywords:** organic DFB laser, amplified spontaneous emission ASE, (co-)polymers, F8BT, surface emitter, diffraction grating

## Abstract

The existence of amplified spontaneous emission (ASE) is a fundamental principle of laser dyes. ASE indicates the spectral variation of the optical gain of a laser dye. Analyzing the spectral distribution of ASE is important for designing lasers. We demonstrate ASE investigations on planar waveguides made of a (co-)polymer. Similar to organic DFB (distributed feedback) lasers, a line grating allows a partial decoupling of the guided radiation. This decoupled radiation is detected as an indicator of the guided radiation. The diffraction of the radiation is utilized to perform a spectrally selective investigation of the ASE by spatially splitting it. This analysis method reduces the influence of isotropic photoluminescence and allows ASE to be analyzed across its entire spectrum. We were able to observe ASE in F8BT over a range from $\lambda_{ASE,min}$ = 530 nm to $\lambda_{ASE,max}$ = 570 nm and determine ASE threshold power densities lower than $E_{ASE}<$ 2.57 μJ/cm^2^. The study of the power density of the ASE threshold is performed spectrally selectively.

## 1. Introduction

Organic semiconductor materials and their wide range of applications have attracted undivided interest in laser research since the first demonstration of amplified spontaneous emission (ASE) in organic thin films [1,2]. Intensive studies and research have enabled lasing with these new laser dyes in a wide variety of resonator architectures [3,4,5,6]. The possibility of lasers with emission wavelengths in the entire visible range and beyond is just one of the advantages [7,8,9,10]. The use of polymers instead of inorganic emitter materials reduces manufacturing effort and therefore costs enormously [9,10]. Simple processes such as spin coating, spray coating or dipping can be used. As there is no crystalline structure, there are also no major requirements for other functional materials in terms of integrability. In addition to these properties, which make conjugated molecules particularly handy, they are characterized by high photoluminescence quantum efficiency and a large stimulated emission cross-section [1,11,12].

These properties have a positive effect on the efficiency of organic lasers, which is characterized by low thresholds. The emission caused by spontaneous emission and amplification by stimulated emission is referred to as amplified spontaneous emission (ASE). ASE is a ubiquitous phenomenon that occurs in any optical system with amplification [13]. From a laser’s point of view, it is important that the presence of losses in a medium results in a threshold in ASE. Overcoming the ASE threshold postulates that spontaneous emission within a pumped sample undergoes a spontaneous amplification greater than the losses. Therefore, a small threshold is an indicator of high relative gain. For studies of laser dyes, a change in threshold can be used to monitor relative changes in net gain [14,15,16]. Knowledge of the photonic band structure of the material is advantageous for use as a laser dye. The spectral dependence of the ASE can help to estimate the efficiency of specific energy level transitions.

A sample arrangement as a planar waveguide and edge emitter is widely used in investigations of laser dyes and ASE. This arrangement is particularly useful for inorganic samples, as sample production is simple and a planar edge is easy to realize in crystalline structures. This method was largely adopted for organic emitters [17,18,19,20,21]. In practice, this approach has a few disadvantages for organic (co-)polymers. Due to the amorphous structure of the polymer films, edge emitters are difficult to manufacture reproducibly. Sample layers do not exhibit a brittle fracture but are plastically deformed and tear. Their decoupling properties can differ. It is also difficult to encapsulate an edge-emitting layer. Photooxidation of (co-)polymers changes their physical and electronic structure by damaging the chain bonds and leads to a dramatic reduction in photoluminescence efficiency [22,23]. Examining a sample without encapsulation can therefore not only be difficult but also affect the results. A difficult work-around is to set up an experiment without oxygen in an inert nitrogen atmosphere or in a vacuum chamber [17]. Encapsulating the organic layer to shield it from the surrounding oxygen is a simple remedy [24,25]. Combining this with edge emission, however, is difficult in practice. The refractive indices of (co-)polymers are smaller than those of inorganic semiconductors. Therefore, a waveguide must be thinner, which causes diffraction effects at the edge emission. The need for detection at small distances results in a high weighting of radiation that is emitted isotropically.

To overcome these drawbacks, a more complex sample design can provide a remedy. Similar to a waveguide attenuation measurement, it is possible to use decoupled scattering losses at the optical interfaces of a waveguide as a measure of the guided intensity. Annavarapu et al. [26] demonstrated that this is possible by using an imaging spectrometer to measure the gain of a planar waveguide.

Here, we present a modification of this method. Instead of detecting scattering losses, an optical diffraction grating is inserted into the interface of the planar waveguide. With appropriate dimensioning, this diffraction grating can decouple part of the intensity guided in the waveguide. An advantage here is a spectral correlation of the decoupled emission and the decoupling direction. Guided intensities of different wavelengths have different optical paths and are therefore diffracted out of the waveguide in different directions [27,28]. Using this novel approach allows for finding the spectral dependence of ASE as well as corresponding thresholds for optically active (co-)polymers without the need for edge-emitting sample geometries.

## 2. Experimental Section

**Figure 1:**
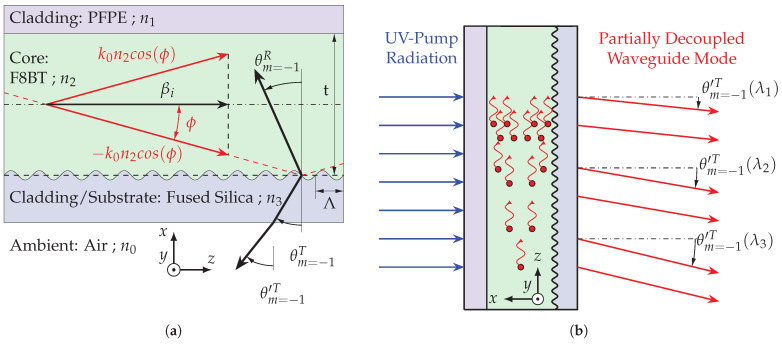
Sample design: (**a**) a cross-sectional view of the organic thin-film sample (not to scale). It consists of a sandwich structure comprising an encapsulation layer (PFPE), a core layer (F8BT) and a substrate (fused silica). A one-dimensional relief grating with periodicity Λ is etched into the surface of the substrate. The plane wave propagation is outlined by the wave vectors (red) and their tangential propagation constant β. The wave vectors of a diffracted wave on the line grating are illustrated in transmitted $k_{m=-1}^{T}$ and reflected $k_{m=-1}^{R}$ diffraction direction for the first diffraction order (black); (**b**) a cross-sectional view of the sample, UV pump radiation and sample emission by partial decoupling of waveguide modes. The figure also contains an illustration of the intensity growth by stimulated emission inside the waveguide.

To investigate the ASE of a (co-)polymer, a thin film sample is constructed. The sample acts as a monomode waveguide for the ASE wavelength band. The waveguide contains a diffraction grating that allows for distributed decoupling of the guided radiation. Elliptically shaped optical excitation of the (co-)polymer favors intensity growth along the long axis (see Figure 1b *z*-axis). The discrete propagation direction in the form of a mode in the waveguide also results in discrete diffraction of the mode and partial decoupling of the mode. The wavelength dependence of the propagation constant of a mode and its diffraction allows a mode-selective investigation based on the decoupling angle.

The sample geometry is similar to an organic DFB laser. Fortunately, the resonant orders of a DFB resonator have a greater spectral distance to each other than the bandwidth of the ASE of most organic (co-)polymers. Thus, it is possible to combine a resonator with a laser dye which exhibits gain only outside of the resonances. Such a non-resonant design of a laser is the design goal for our samples.

### 2.1. Sample Design

Figure 1 shows a cross-section of the sample layout. Plane waves propagating in the core layer (outlined by their wave vectors) can be guided by reflection at the interfaces to the cladding layers in the waveguide. The waveguide consists of three layers and is assembled in a sandwich structure. The core layer is made of (co-)polymer F8BT and has a refractive index of $n_{2}$. To ensure total internal reflection at the interfaces, the surrounding cladding layers must each have a lower refractive index. Therefore, the substrate consists of fused silica with a refractive index of $n_{3}$ and the cladding layer above the core layer is a perfluoropolyether (PFPE) with a refractive index of $n_{1}$.

The transport of energy in the waveguide is by waveguide modes, which are a representation of the static field distribution in the waveguide. The field of the waveguide results from the superposition of the waves propagating in the waveguide. To obtain a stationary field, a propagating plane wave must be in phase with itself after one cycle to reproduce itself. Propagation constant $\beta_{i}$ of a mode describes the direction of propagation of a planar wave that represents a waveguide mode:

(1) $$\beta_{i}=n_{2}k_{0}\cdot cos\left(\phi_{i}\right),$$

where $k_{0}$ is the wavenumber $\left(k_{0}=2\pi /\lambda \right)$ and $\phi_{i}$ is the angle between the boundary and the propagation direction. In order to obtain a stationary field for a waveguide, only discrete propagation constants are possible. For a constant wavelength, the number of discrete permissible propagation directions increases with increasing waveguide thickness.

Guided radiation confined in the core layer assumes different modes due to the different distributions of the electric and magnetic field components. These field modes represent different paths that the radiation can propagate through the waveguide while wavelength and polarization satisfy the boundary conditions of the waveguide. The propagation constants for a waveguide as a function of wavelength can consequently be characterized on the basis of the resulting field.

The wave equation must be solved to determine the field. Under the condition that the field only changes along the x coordinate, the wave equation can be set up separately for each area of the waveguide. The solution of the estimated field E(x) occurs in an exponential decay in the cladding layers, while a sinusoidal characteristic is required in the core layer. The continuous field distribution across the layer interfaces leads to eigenvalue Problem (2) whose solutions are the propagation constants.

(2) $$tan\left(t\cdot \sqrt{k^{2}n_{2}^{2}-\beta_{i}^{2}}\right)=\frac{\sqrt{\beta_{i}^{2}-k^{2}n_{1}^{2}}+\sqrt{\beta_{i}^{2}-k^{2}n_{3}^{2}}}{\sqrt{k^{2}n_{2}^{2}-\beta_{i}^{2}}-\frac{\sqrt{\beta_{i}^{2}-k^{2}n_{1}^{2}}\cdot \sqrt{\beta_{i}^{2}-k^{2}n_{3}^{2}}}{\sqrt{k^{2}n_{2}^{2}-\beta_{i}^{2}}}}$$

**Figure 2:**
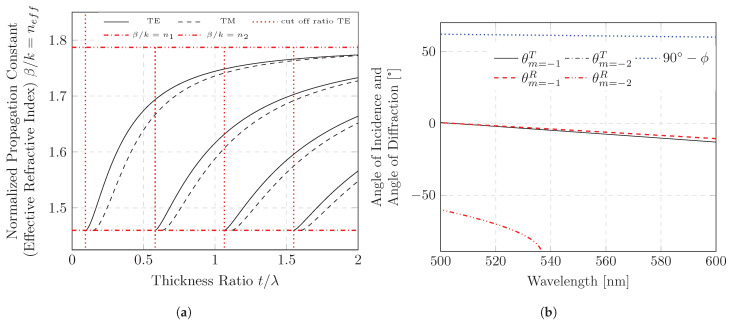
Numerical estimation of wave propagation using constant refractive indices ($n_{1}$ = 1.275; $n_{2}$ = 1.787; $n_{3}$ = 1.459): (**a**) dispersion curve: normalized propagation constant β/k as a function of the ratio of core layer thickness and wavelength, determined by solving eigenvalue Problem (2); (**b**) wave propagation angle at the boundary to optical grating. Angle in the projection surface of the grating vector and the *z* axis, calculated by solving eigenvalue Problem (2) and discrete diffraction at the grating (4) for a first-order transverse electric mode (TE00) and a waveguide with a constant layer thickness *t* = 137.5 nm (t/λ = 0.25 at λ = 550 nm) and constant refractive indices as a function of wavelength. Diffraction by a grating with a periodicity of Λ = 320 nm.

The eigenvalue problem in Equation (2) [29] shows the relationship between the propagation constant and the waveguide of a transverse electric wave. In addition to geometrical and material properties such as the refractive indices of the layers and the core layer thickness, propagation depends only on the wavelength. Propagation constants can be estimated numerically. Figure 2a is a plot of the wavelength-normalized propagation constant $\beta_{i}$ as a function of the layer thickness ratio t/λ. The wavelength-normalized propagation constant $\beta /k_{0}$ is often also referred to as the effective refractive index $n_{eff}$. In addition to transverse electric waves (TE), transverse magnetic waves (TM) can also propagate in the waveguide. Their propagation constants are also plotted as a function of thickness ratio in Figure 2a. The propagation constants of the TM modes can also be predicted on the basis of a phase condition. However, the phase jump at the interfaces differs due to the polarization and a mode with the same wavelength results in a smaller effective refractive index. Due to polarization, the reflection of these waves is less efficient at the waveguide interfaces, which is why they are negligible compared to the TE waves in our measurement. Due to the laser-like sample geometry, a possible resonance should be taken into account when designing the sample. As highlighted in Figure 2a (dotted cut-off ratio), there is a minimum thickness ratio for each propagation order below which the propagation order disappears. To realize a first-order waveguide, the ratio of layer thickness to wavelength must not exceed the cut-off ratio of the second order.

In order to obtain a large difference in wavelength between TE and TM modes, a layer thickness ratio of less than 0.4 is desired. Using a constant layer thickness, the change in the propagation constant can be determined as a function of wavelength. The propagation direction of the waves based on their propagation angle to the interface (ϕ) can be determined using Equation (1) and are plotted as a function of wavelength in Figure 2b. The considered emission range is symmetrical around the spectral ASE maximum $\lambda_{ASEpeak}$ = 560 nm [25,30] of the (co-)polymer F8BT used in this study and should extend far beyond the full width half-maximum (FWHM) limits of ±15–20 nm.

Diffraction grating, which has so far been neglected in the description of mode propagation, can be used to partially decouple a guided wave. Based on the fact that waves in periodic structures assume the same periodicity as their host, partial waves are diffracted in discrete directions. A plane wave transmitted or reflected through a periodic grating also results in a periodic wavefront and transmits the power in several discrete directions which are referred to as diffraction orders.

(3) $$n_{2,3}\cdot sin\left(\theta_{m}^{r,t}\right)=n_{2}\cdot cos\left(\phi \right)+\frac{m\cdot \lambda_{0}}{\Lambda },$$

The direction of these discrete diffraction orders can be described by grating Equation (3), where *m* describes the order of diffraction, Λ is the periodicity of the grating, ϕ is the angle between the incident plane wave and the grating boundary and is $\theta_{m}^{r,t}$ the respective angle of the diffracted order for reflected or transmitted direction to the surface normal. The diffraction properties depend on the wavelength, the periodicity of the grating and the direction of the guided wave. Diffraction occurs in reflection and transmission. Diffraction orders in transmission propagate in the grating substrate and diffraction orders in reflection propagate in the core layer. Taking into account the critical angles, diffraction orders can propagate out of the waveguide structure. Figure 1 outlines the first diffraction orders by their wave vectors for transmission and reflection direction.

In addition to partial decoupling, the diffraction orders can also cause resonance. To generate a resonance for a DFB resonator, part of the wave must be diffracted back in the direction from which the incident wave originates. For the special case ($|\theta_{m}^{r}|+|\phi_{i}nc|=\pi /2$), diffraction Equation (3) becomes the well-known Bragg equation.

(4) $$2\cdot n_{2}\cdot cos\left(\phi \right)=2\cdot n_{eff}=\frac{m\cdot \lambda_{Bragg}}{\Lambda }$$

Resonance for the guided mode is enabled in close vicinity of the Bragg wavelength ($\lambda_{Bragg}$) given by the Bragg condition [8,31]. Resonance can occur for any order in this form. A DFB resonator is distinguished by the order that leads to feedback. For instance, a DFB resonator of the first order provides feedback through the first diffraction order, while a DFB resonator of the second order provides feedback through the second diffraction order and so on. The efficiency of feedback decreases with higher orders [31,32], which is why lasing requires higher amplification.

In this work, we do not want to focus on resonant diffraction orders but on a behavior that explicitly does not allow resonance. The sample set up is a DFB resonator in which the grating periodicity is too small to enable resonance of the second DFB order and too large to enable resonance of the first DFB order for ASE. In this case, the first diffraction order can partially decouple the guided mode. Figure 2b displays the diffraction angles as a function of wavelength for a sample geometry constructed from these perspectives. The Bragg wavelength of a second-order DFB resonator is located at the left edge of the diagram, with a wavelength of 504.25 nm. In this spectral range, amplification from our employed (co-)polymer F8BT is unlikely. The angle of the first diffraction order (which we use for our measurements) varies as a function of wavelength in a nearly linear behavior. The diffraction angle of the first diffraction orders does not exceed that of the critical angle and it is close to the surface normal.

### 2.2. Fabrication

A schematic cross-section of the device is shown in Figure 1. The organic emission layer and an encapsulation layer are stacked on a patterned fused silica produced by laser interference lithography, which from now on is denoted as substrate. The substrate carries various sinusoidal one-dimensional gratings with periods from Λ = 280 nm to Λ = 420 nm. The active organic material consists of a ratio-optimized F8BT, a commercially available (co-)polymer of 9,9-dioctylfluorene (F8) and benzothiadiazole (BT) (ADS233YE; American Dye Source, Inc., Baie d’Urfé, QC, Canada). The adjusted F8:BT ratio of 9:1 leads to a lower ASE threshold power density compared to regular F8BT [33]. A low threshold power density of 4 μJ/cm^2^ at λ = 560 nm [30] qualifies the (co-)polymer as the preferred choice of dye for applications requiring high efficiency [9,10,25]. F8BT is dissolved in toluene at a concentration of 30 mg/mL. In a spin coating process, the active organic material is applied to the substrate at a spin speed of 2000 rpm in a toluene-saturated atmosphere. This results in an approx. 144 nm thick amorphous layer that forms the optical waveguide. To reduce photo-oxidation and increase the life expectancy of sample emission, the organic layer is encapsulated with a PFPE-urethane methacrylate layer (Fluorolink^®^ MD700; Solvay GmbH, Hannover, Germany) and a cover slip. All layers have a high transmittance in the visible spectrum and thus emission from the polymer film can be radiated in bottom and top directions.

### 2.3. Optical Setup

**Figure 3:**
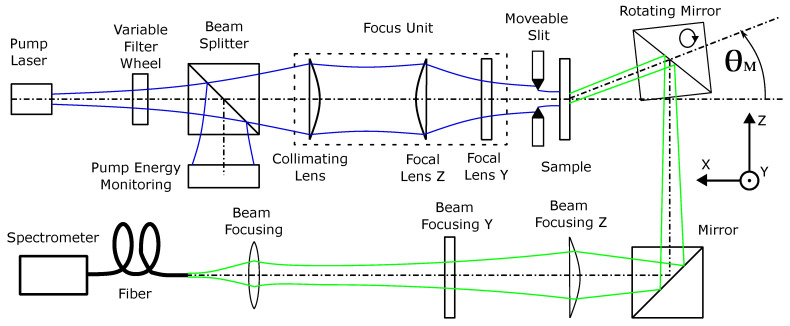
Schematic representation of the optical pump set-up (including the focus unit) and the emission measurement set-up for an organic sample with diffracted angular emission. A rotatable mirror is used to detect radiation that deviates from the surface normal of the sample. The angle of measurement, denoted as $\theta_{M}$, enables selective detection of modes by restricting the range of emission angles.

Figure 3 outlines the optical pump setup and emission measurement setup of the organic thin-film sample in our laboratory. Sketched in blue is the UV pump radiation required to optically pump the organic sample. The pump pulse of a frequency-tripled passively Q-switched ND:YAG laser (FTSS355-Q2; CryLaS, Berlin, Germany)) with a wavelength of $\lambda_{pump}$ = 355 nm exhibits a pulse duration of 1.9 ns. Unless otherwise specified, all measurements are carried out at a pump pulse repetition rate of 1 kHz. The use of neutral density (ND) filters in the form of a variable ND filter mounted on a stage allows variation of the pump energy. A beam splitter (92:8) was positioned in the pump beam behind the ND filters for pump pulse energy monitoring. Pump energy monitoring was performed using an energy sensor (Pe10b; Gentec-EO, Quebec City, QC, Canada). A focusing unit consisting of a collimator and two plano-convex lenses was used to create an elliptical pump spot with beam diameter in length ($\omega_{z}$ = 996 μm) and diameter in width ($\omega_{x}$ = 286 μm). To achieve a homogeneous pump power density along the pump stripe, two movable razor blades were arranged in the pump beam. The blades were positioned at a small distance (<500 μm) in front of the sample to reduce diffraction effects. The distance between the blades was similar to the beam diameter ($\omega_{y}$) and the length of the pump stripe resulted in a top hat profile with a length of $l_{z}$ = 830 μm. The organic sample was arranged with the surface normal parallel to the pump beam. The periodic line grating resulted in an alternating refractive index in the z direction. The organic sample emission is outlined in green in Figure 3. Depending on the grating period, the emission of the sample was diffracted at an angle $\theta_{M}$ to the normal of the sample surface. To detect this angle-dependent emission, a rotatable mirror was located behind the sample. The detection of the organic sample emission was performed with a fiber-coupled spectrometer (USB2000+; OceanOptics, Orlando, FL, USA). To differentiate between TE and TM radiation, a polarizer was inserted into the beam path of the sample emission if required. Fiber coupling of radiation parallel to the measurement axis was achieved by a Kepler telescope. It consists of two plano-convex cylindrical lenses as z and y objectives and a biconvex converging lens as ocular.

## 3. Results

**Figure 4:**
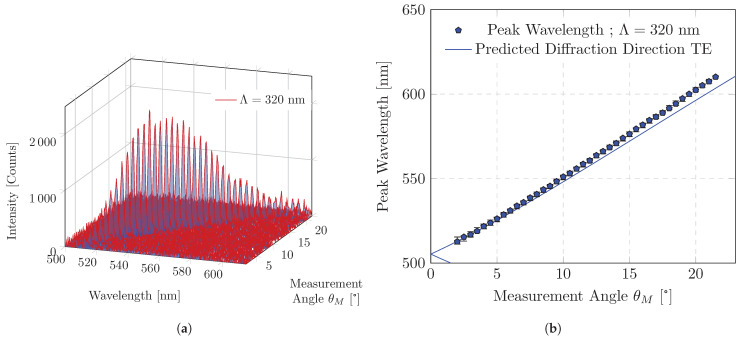
Emission spectrum as a function of measurement angle $\theta_{M}$: (**a**) The measured signal results from partial first-order diffraction of guided modes propagating from bottom to top through the waveguide; (**b**) Peak emission wavelength of the emission spectrum as a function of measurement angle $\theta_{M}$. Error bars indicate the FWHM limits of the emission spectrum. Also shown is a numerical prediction of the mode wavelength as a function of diffraction direction $\theta {’}_{m=-1}^{T}$ using wavelength-dependent refractive indices.

The presented results are divided into spectral and direction behavior as well as ASE threshold determination. The isotropy of the spontaneous emission enables excitation equally in all spatial directions. The waveguide and the line-shaped excitation favor a single spatial direction along the z coordinate. Mode propagation in the positive direction along the z axis from bottom to top through the sample must always be accompanied by a contra-directional mode propagation in the negative direction along the z axis from top to bottom due to superposition. The partial diffraction of a mode in the direction of diffraction angle $\theta^{\prime }$ (see Figure 4a) therefore occurs symmetrically with a diffraction angle of an inverted sign. To investigate the ASE emission, the partial decoupling of the mode propagating upwards in the sample is used, as illustrated in Figure 1b and Figure 3.

### 3.1. Diffraction Direction and Surface Emission Spectrum

**Figure 5:**
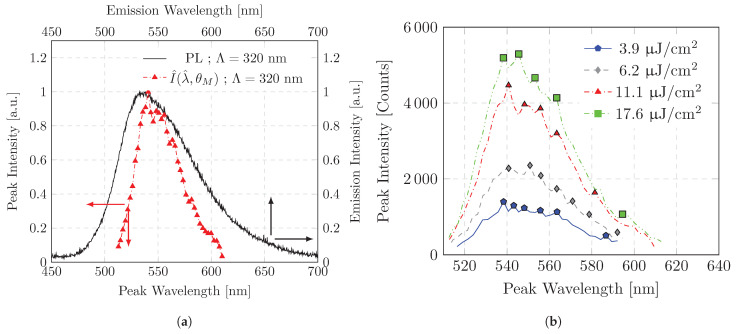
Emission characteristics of a sample that does not support distributed feedback in the spectral range of the ASE: (**a**) Photoluminescence spectrum measured in the direction of the surface normal and peak emission intensities of emission spectra as a function of peak emission wavelengths of a step by step measurement; (**b**) Peak emission intensities of emission spectra as a function of peak emission wavelengths of a step-by-step measurement. Measured with a variety of pump energy densities. Marks indicate local maximas of the spectral band.

The investigation of the ASE on the basis of a partial surface emission presupposes the absence of resonance. Therefore, a sample with a periodicity that is too small to allow a second-order TE DFB resonance is used. To exclude a sample with second-order resonance, surface normal emission was investigated. The photoluminescence spectrum is shown in Figure 5a. The photoluminescence spectrum has an intensity peak at $\lambda_{PL,max}$ = 532 nm and exhibits a broad emission spectrum from 509 nm to 589 nm (FWHM). It is clear that the photoluminescence spectrum is almost identical to a photoluminescence spectrum of a neat F8BT film without a corrugation [25]. For a second-order DFB laser, Bragg scattering through the first diffraction order would drastically modify the emission spectrum. A narrowband peak would be expected in the spectrum [27,28]. This emission behavior could be observed for longer grating periodicities on the sample (Λ = 340 nm to 380 nm).

A plane wave can propagate in the waveguide and is partially decoupled through the grating. The direction of decoupling correlates with the wavelength. To detect the decoupled emission, the spectrum must be measured as a function of the diffraction angle. The examination of a spectral band is conducted in a stepwise manner for a range of discrete measurement angles. Figure 4a shows the spectra of such a measurement as a function of the angle of measurement. The sample was excited with a constant excitation power density during the measurement and the measurement angle was changed in steps of 0.5°. The average FWHM of the emission peak is ${\bar{\Delta \lambda }}_{\Lambda =320nm}$ = 2.6 nm, and no significant change was observed for the range of measurement angles. To assign a modal wavelength, the peak emission wavelengths are calculated by fitting a normal distribution for each emission spectra and plotted as a function of the measurement angle in Figure 4b. The peak wavelength changes in an almost linear correlation of the measurement angle. This behavior is expected for small changes in the emission wavelength with respect to the thickness of the waveguide (λ/t≈0.23 to 0.27) and for small measurement angles. For large changes in the wavelength relative to the layer thickness, there is a large change in the layer thickness ratio. The non-linear relationship between the propagation constant and the mode wavelength should then be more weighted and result in an equally non-linear decoupling (see Figure 2a). It should also be noted that the diffracted mode is refracted at the interface to the ambient during decoupling. A linear behavior ($sin\left(x\right)\approx x$) can only be approximated for small measurement angles.

Figure 4b also contains a propagation prediction of the first transmitted diffraction mode of TE modes, as indicated by the solid line. This prediction was obtained from a numerical solution of waveguide eigenvalue Problem (2) and diffraction Equation (3). The behavior of waveguide modes in (co-)polymers is complicated by the fact that the refractive index varies with wavelength and layer thickness [34]. Published ellipsometry refractive indices were considered for the encapsulation layers [35,36]. The refractive index of the (co-)polymer is determined using resonance wavelengths of longer grating periodicities of the sample, eigenvalue Problem (2) and Bragg Equation (4) (see Table A1). This takes an approximate account of the (co-)polymer layer thickness.

A spectrum similar to the ASE one should be expected when the measured peak intensity is plotted as a function of its peak wavelength (see Figure 5a,b). Modes amplified by stimulated emission should become more prominent than unamplified modes in terms of their emission wavelength. The emission spectrum of the modal decoupled intensity has a smaller bandwidth compared to the photoluminescence spectrum. The spectrum emitted by the sample can be localized at a peak wavelength of $\lambda_{ASE,Peak}\approx$ 545 nm and has a FWHM of 47–49 nm. The spectrum has a positive skew and appears to be composed of several spectral peaks. In comparison to published data in neat films, the spectrum is similar but more broadband, with peak emission wavelength $\lambda_{ASE,Peak}\approx$ 560 nm with an FWHM of 15–20 nm [25,30].

An examination of the emission spectrum suggests that it is composed of several narrowband emission peaks. The highlighting of local maxima in the spectrum indicates the presence of a variety of local peaks (indicated by marks in Figure 5b). The local maxima may represent discrete energy transitions of the (co-)polymer. It is notable that not all peaks at different excitation power densities have identical emission wavelengths. This could be due to spectral resolution restraints of the measurement. The spectral resolution correlates approximately with the stepwise change in the measurement angle and is on average $\Delta \lambda_{Peak}\approx$ 2.5 nm (spectral distance between the peak emission wavelengths; see Figure 4b).

### 3.2. ASE Threshold

To prove the occurrence of ASE, the spectral bandwidth as a function of pump excitation power density is often considered. Above the ASE threshold, the ASE intensity increases more than the photoluminescence (PL). The bandwidth is narrowed with increasing excitation. This technique cannot be used here as the bandwidth is already selected due to the acceptance angle of the sample emission measurement set up.

**Figure 6:**
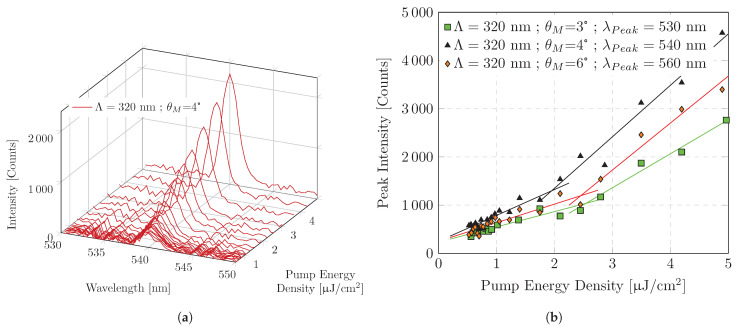
ASE threshold measurement for various emission wavelengths: (**a**) Emission intensity as a function of emission wavelength and excitation pump energy density for one selected measurement angle $\theta_{M}$; (**b**) ASE threshold determination for three selected emission wavelengths. Measurement angle $\theta_{M}$ results in peak emission wavelength $\lambda_{Peak}$. Peak emission intensity growth for three different measurement angles and therefore three different emission wavelengths as a function of pump energy density. Also shown are linear functions estimated by linear regression below and above a predicted threshold.

Another option is to determine the ASE threshold by intensity growth. The intensity of the ASE as a function of the pump power density must have a threshold behavior. This method is most suitable for our sample constellation. The spatial separation of the spectral emission as a function of the diffraction angle allows a wavelength-dependent analysis. Due to the distributed decoupling, a high ASE to PL ratio can be obtained. Guided mode decoupling results in diffraction modes that can be separated using geometric optics. The emission intensity as a function of wavelength and as a function of pump energy density in Figure 6a shows that the measurement results in a constant peak emission wavelength and FWHM. This measurement is performed for different emission wavelengths, using the intensity of the peak wavelength resulting from a diffracted signal using a constant angle $\theta_{M}$ during the measurement. Only intensities characterized by a constant peak wavelength (Δλ=±0.1 nm) have been used for threshold determination.

**Figure 7:**
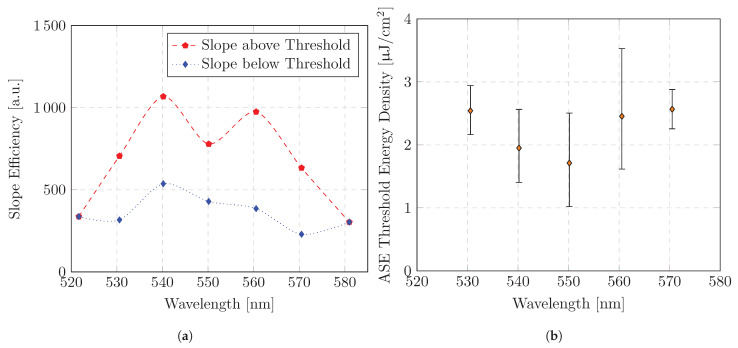
ASE threshold and emissionsion intensity slope for various emission wavelengths: (**a**) Slope efficiency of the peak emission intensity above and below the ASE threshold power density as a function of the emission wavelength. Determined by linear regression. (**b**) ASE threshold power density as a function of the emission wavelength.

The ASE threshold can be identified from the function curve of the peak emission intensity as a function of the excitation power density (Figure 6b). Significant thresholds are found for an emission range from $\lambda_{min}$ = 530 nm to $\lambda_{max}$ = 570 nm. In order to obtain values for the ASE threshold, two linear regressions are calculated for the intensity growth. The two linear regressions describe the intensity growth above and below the ASE threshold, respectively. The ASE threshold calculated from the intercepts of the linear regressions shows a minimum for emission range λ = 540 nm and λ = 550 nm (Figure 7b). The threshold increases for longer and shorter emission wavelengths. As can be seen in Figure 7b, the calculated ASE thresholds are consistently less than 2.57 μJ/cm^2^. In Figure 7a, in addition to the ASE threshold, the slope efficiency of the emission intensity above and below the threshold is plotted as a function of emission wavelength. Above the threshold, emission growth shows linear behavior. In the investigated range up to approx. 20 μJ/cm^2^, no saturation behavior can be observed.

## 4. Discussion

The ASE spectrum and the ASE threshold can be determined via partial decoupling of the guided waveguide modes. This method has the advantage of providing each decoupled waveguide mode with a different spatial propagation direction. Using simple geometrical optics, it is possible to examine waveguide modes with different wavelengths separately. A wavelength-selective investigation of the ASE appears to provide information about the efficiency of stimulated energy transition. This classification system can be used not only for optically amplifying materials but also for identifying potential pump sources. The principal challenge associated with the partial decoupling method pertains to the intricate sample design. It is imperative to calibrate the layer thickness of the active layer with a diffraction grating while accounting for the refractive indices of the waveguide layers. A limiting factor in the investigation is the thin layers of active dye required, which necessitate high absorption during optical pumping. The methodology presented is not suitable for investigating gains in dyes. However, it is anticipated that future investigations of the partial decoupling method will allow for additional measurements of the gain through a near-field analysis of the decoupled radiation.

Distributed decoupling allows greater emphasis to be placed on the emission intensity generated by the modes. Stimulated emission is observed to result in an intensity growth phenomenon in a preferred spatial direction. The common edge emission method tends to privilege the isotropic spontaneous emission more in relation to the signal of the waveguide mode. We assume that this is due to the fact that the layer thicknesses are very small and the edge emission causes a strong divergence of the outcoupled radiation. This is why focusing optics at small distances are recommended for measurement. As a result, a large proportion of the isotropically emitted radiation is also detected. Compared to the commonly employed variable stripe length method, the ASE measurements reported here using the distributed decoupling method are less superimposed by spontaneous emission and the determined threshold is therefore more accurate. As an example, Samuel et al. [30] reported a peak ASE wavelength that is in good agreement with our results and their reported threshold is just slightly higher, which agrees well with the hypothesis that our results are less superimposed by spontaneous emission.

The shape and position of the measured ASE as a function of wavelength differ from those reported by other studies in the same material [25,30]. To gain a deeper understanding of these differences, we investigated a planar waveguide without corrugation and found ASE surface emission due to scattering. The peak ASE emission wavelength was ≈560 nm and the FWHM was ≈17 nm. Both the waveguide structure and the thickness of the waveguide were fabricated identically. However, the pump energy density was significantly higher for the non-corrugated waveguide in order to differentiate the ASE in the measured signal. This could be an indicator that the pump energy density influences the spectral shape of the ASE, which emphasizes that a selection of the ASE is fundamental for an independent analysis. The differentiated investigation of the intensity growth above and below the ASE threshold shows spectral characteristics of spontaneous and stimulated emission. The emission below the ASE threshold is more strongly weighted by spontaneous emission. Above the ASE threshold, emission is more strongly weighted by stimulated emission. The slope efficiency above the ASE threshold can be interpreted as the relative increase in net gain as a function of pump power density. The slope efficiency above the ASE threshold shows two local maxima across the ASE range. Below the ASE threshold, one of these local maxima nearly disappears (at λ=560 nm). The observed increase in gain for this emission range is presumed to be primarily attributable to stimulated emission. This implies that a high amplification above the ASE threshold can be expected. At the second local maximum (at λ=540 nm), however, the behavior is different. The discrepancy in slope efficiency above and below the ASE threshold is relatively minor. This indicates a more pronounced spontaneous emission, which results in a lower threshold.

In order to generate an amplified resonance with an optical amplifier, it is helpful to examine both spontaneous and stimulated emission. Depending on the application, a low ASE threshold may be advantageous over a strong stimulated emission.

## 5. Conclusions

In this work, we demonstrate ASE investigations on planar waveguides of (co-)polymer F8BT. In order to improve the detection of the spectral distribution of the ASE, the partially decoupled radiation from a line grating is detected as an indicator of the guided radiation. The diffraction of the radiation is utilized to perform a spectrally selective investigation of the ASE by spatially splitting it. This analysis method reduces the influence of isotropic photoluminescence and allows for ASE to be analyzed across its entire spectrum. Furthermore, we note that this method can be used for basically any optically active polymer films, including composite polymers. We observe ASE in F8BT over a range from $\lambda_{ASE,min}$ = 530 nm to $\lambda_{ASE,max}=$ 570 nm and determine spectrally selective ASE threshold power densities lower than $E_{ASE}<$ 2.57 μJ/cm^2^. 

## Data Availability

The original contributions presented in the study are included in the article, further inquiries can be directed to the corresponding author.

## Appendix A. Refractive Index F8BT

The refractive index of the (co-)polymer is determined using resonance wavelengths of longer grating periodicities of the sample, eigenvalue Problem (2) and Bragg Equation (4). This takes an approximate account of the (co-)polymer layer thickness.

**Table A1:** Calculated refractive index of core layer. Layer thickness *t* = 144 nm.

Periodicity	Bragg Wavelength	Reffractive Index
Λ [nm]	$\lambda_{\mathrm{Bragg}}$ [nm]	$n_{2}$
330	516	1.7883
340	527.9	1.7781
350	540.4	1.7706
360	553.1	1.7648
370	566.1	1.7606
380	580.4	1.7630
